# Hsp90β promoted endothelial cell-dependent tumor angiogenesis in hepatocellular carcinoma

**DOI:** 10.1186/s12943-017-0640-9

**Published:** 2017-03-31

**Authors:** Jing Meng, Yanrong Liu, Jingxia Han, Qiang Tan, Shuang Chen, Kailiang Qiao, Honggang Zhou, Tao Sun, Cheng Yang

**Affiliations:** 1 State Key Laboratory of Medicinal Chemical Biology and College of Pharmacy, Nankai University, Tianjin, 300350 China; 2 Tianjin Key Laboratory of Molecular Drug Research, Tianjin International Joint Academy of Biomedicine, Tianjin, China

**Keywords:** Hsp90β, Angiogenesis, NVP-BEP800, Endothelial cell, Hepatocellular carcinoma

## Abstract

**Background:**

Vascular endothelial growth factor receptors (VEGFRs) are the major receptors involved in endothelial cell-dependent tumor angiogenesis. There are studies account for the effects of Hsp90 on angiogenesis, but the role and mechanism of Hsp90β isoforms and NVP-BEP800, a specific inhibitor of Hsp90β, in tumor angiogenesis is rarely mentioned.

**Methods:**

Immunohistochemistry and statistical analysis was used to evaluate the correlation between Hsp90β expression, CD31 endothelial cell-dependent vessel density, and VEGFRs expression in tissue samples of 96 HCCs. Kaplan-Meier survival analysis and COX proportional hazards analysis the relation of Hsp90β and prognosis. HUVEC cells were transfected with Hsp90β or treated with NVP-BEP800, and then cell proliferation, migration, invasion and tube formation were investigated. The VEGFR1 and VEGFR2 expression was determined by Western blot and immunofluorescence. The VEGFR1 and VEGFR2 promoter activities were detected by dual luciferase report system. In vivo, the angiogenesis promotion of Hsp90β and anti-angiogenesis efficacy of NVP-BEP800 was tested in HCC xenograft models. Histological analysis was performed on tumor samples to evaluate Hsp90β, VEGFRs expression and MVD.

**Results:**

This study investigated the correlation between Hsp90β expression and CD31+ endothelial cell-dependent vessel density. Hsp90β promoted VEGFRs expression by increasing their promoter activities. The proliferation, migration, invasion, and tube formation activities of human endothelial cells significantly increased when Hsp90β was overexpressed. NVP-BEP800 down-regulated VEGFRs expression to significantly reduce tubular differentiation, as well as endothelial cell proliferation, migration, and invasion. Furthermore, NVP-BEP800 decreased VEGFR1 and VEGFR2 promoter activities. In vivo, Hsp90β promoted VEGFRs and CD31 expression in human hepatocellular carcinoma tumor xenografts and was associated with increased tumor microvessel density. After 18 days of treatment with 30 mg/kg/day NVP-BEP800, VEGFRs and CD31 expression significantly decreased.

**Conclusion:**

Hsp90β induced endothelial cell-dependent tumor angiogenesis by activating VEGFRs transcription. NVP-BEP800 has potential as a therapeutic strategy for inhibiting tumor angiogenesis by decreasing endothelial cell progression and metastasis. It can help develop a therapeutic strategy for tumor treatment through the inhibition of endothelial cell progression and metastasis.

**Electronic supplementary material:**

The online version of this article (doi:10.1186/s12943-017-0640-9) contains supplementary material, which is available to authorized users.

## Background

Tumor initiation and malignant evolution are closely related to angiogenesis [1, 2]. A physiological or pathological neovascularization process is necessary to initiate tumor tissue ischemia, growth, or metastasis [3]. In angiogenesis, endothelial cell proliferation and migration cause new capillaries to develop from preexisting capillaries [4]. In addition, tumor cells, pericytes, immune cells, hematopoietic cells, circulating endothelial progenitor cells, and various types of molecules are also involved in the complicated process of angiogenesis [5]. Angiogenesis involves complex signaling pathways and many key regulatory factors, such as vascular endothelial growth factor (VEGF)/vascular endothelial growth factor receptor (VEGFR) [6–8], angiopoietins, integrins, neuropilin-1, thrombospondin-1, vasohibin, and NOTCH/delta-like protein 4 pathway [5]. Among these pathways, the VEGF/VEGFR system of endothelial cells is the classical pathway used in blood vessel formation [9, 10]. The VEGF receptor family includes VEGFR1, VEGFR2, and VEGFR3 [11]. The high affinity of VEGF and VEGFR1, VEGFR2 induce proliferation, migration and differentiation of vascular endothelial cells [12]. Activated endothelial cells degrade the extracellular matrix, subsequently form tubular structures, and recruite supporting cells to form stable vessels [1, 13, 14]. Therefore, VEGF/VEGFR is an crucial pathway in angiogenesis of tumor.

Heat shock protein 90 (Hsp90) is a molecular chaperone that is induced in response to cellular stress. Hsp90 stabilizes client proteins involved in the cell cycle, proliferation, migration, and apoptosis [15]. The Hsp90 family has four major members: Hsp90α, Hsp90β, GRP94, and Hsp75. Evidence suggest that Hsp90 is important in regulating tumor angiogenesis. Hsp90, as a key protein chaperone, plays an important role in the PI-3 K/AKT/mTOR complex of the PI-3 K/AKT signaling pathway, which promotes endothelial cell survival [16, 17]. VEGFR2 is also reported to be a client protein of Hsp90, which can form a complex with human umbilical vein endothelial cells (HUVEC) [18]. Furthermore, the Hsp90 inhibitor suppresses the expression of VEGFRs and VEGFR complexes, such as integrins [19], focal adhesion kinase (FAK), and neuropilin coreceptors [5]. Although most studies account for the effects of Hsp90α isoforms on angiogenesis, the role and mechanism of Hsp90β isoforms in tumor angiogenesis is rarely mentioned.

We have previously established that Hsp90β is associated with the tumor malignancy of hepatocellular carcinomas (HCCs). HCC is a highly vascular tumor, thus providing an attractive target for the development of new anticancer drugs is necessary [20]. In the present study, we evaluate whether Hsp90β expression increased in HCCs with a high degree of malignancy. Results showed that there was an obvious relationship between Hsp90β expression and endothelial cell-dependent vessel density. Hsp90β increased VEGFRs expression by activating their promoters and enhancing endothelial cell proliferation, migration, and tubular structure formation. NVP-BEP800, an Hsp90β inhibitor, decreased VEGFRs expression and inhibited HCC tumor angiogenesis. Therefore, NVP-BEP800 has potential as a therapeutic strategy for inhibiting tumor angiogenesis by decreasing endothelial cell progression and metastasis.

## Methods

### Cell culture and transfections

HUVECs and PLC-PRF-5 cells were obtained from the Cell Bank of Shanghai Institute for Biological Sciences (Shanghai, China). HUVEC cells were cultured in medium 199 (M-199) containing endothelial cell growth supplement, 10% fetal bovine serum (FBS), and 1% antibiotics (100 U/mL penicillin and 10 μg/mL streptomycin). PLC-PRF-5 cells were cultured in Roswell Park Memorial Institute 1640 medium supplemented with 10% FBS and penicillin–streptomycin solution. All cells were maintained at 37 °C in a 5% CO_2_ incubator. All the plasmids were transfected into cells by Lipofectamine 2000 (Invitrogen, USA).

### Luciferase activity assays

HUVECs were seeded in 96-well plates. After 24 h, the plasmids, namely, pEZX–PG04–VEGFR1 and pEZX–PG04–VEGFR2 (FulenGene, China), were either transfected separately into cells, co-transfected with pcDNA3–Hsp90β or pRNAT-U6-siHsp90β, or incubated with NVP-BEP800 (Selleck, USA) for 48 h. Gaussia and secreted alkaline phosphatase (SEAP) luciferase activities were consecutively measured by a dual-luciferase reporter assay system (FulenGene, China) after 48 h of transfection. Gaussia luciferase activity was normalized to SEAP activity.

### Cell invasion assays

HUVECs in serum-free medium were seeded onto a chamber coated with Matrigel (BD, USA) and inserted into the wells of a 24-well plate. FBS was added to a 24-well plate located below the chamber to serve as a chemoattractant. After 24 h, invasive cells located on the lower surface of the chamber were stained with 0.1% crystal violet and counted.

### Wound healing assay

HUVECs were seeded at 80% to 90% confluence in 24-well plates. An area of cells was removed with a pipette tip drawn across the center of each well. Cells were washed thrice followed by treatment with FBS-free medium containing DMSO or 2 μM NVP-BEP800. The speed of wound closure was monitored after 16, 32, and 48 h by comparing the wound distance ratio at 0 h. This experiment was independently repeated thrice.

### Tube formation assay

HUVECs were seeded onto a 48-well plate coated with Matrigel (BD, USA) followed by treatment with a medium containing DMSO or 2 μM NVP-BEP800. Peak tube formation was observed at 12 h post-treatment. Four random fields were observed under a microscope. The number of tubes for each treatment was quantified. This experiment was independently repeated thrice.

### Western blot analysis

Cells were washed with phosphate-buffered saline (PBS) and lysed in ice-cold lysis buffer containing protease inhibitor cocktail (Sigma) for 30 min. Lysates were separated by electrophoresis and transferred onto polyvinylidene difluoride membranes (Millipore; Bedford, MA, USA). The membranes were blocked and incubated with the primary antibodies of Hsp90β, VEGFR1, and VEGFR2 (CST, USA). The membranes were then incubated with a goat anti-rabbit IgG–HRP secondary antibody (Thermo Scientific, USA). GAPDH was used as the loading control. Protein expression was assessed with enhanced chemiluminescent substrate (Millipore, USA) and by exposure to chemiluminescent film.

### Immunofluorescence

HUVECs were fixed with 3.7% formaldehyde in PBS for 5 min at room temperature and blocked with 1% bovine serum albumin for 30 min. The cells were incubated overnight at 4 °C with VEGFR1 antibody (1:200; CST, USA) or VEGFR2 antibody (1:200, CST, USA), and then incubated with FITC- and TRITC-labeled secondary antibodies (Earthox LLC, USA) for 1 h at room temperature. Each step was followed by two 5-min washes in PBS. The prepared specimens were counterstained with DAPI (Southern Biotechnology Associates Inc., USA) for 2 min and observed with a confocal microscope (Nikon, Japan).

### Immunohistochemistry assay and analysis

Paraffin sections of human samples and tumor tissue of mice were deparaffinized in xylene and rehydrated in a series of graded alcohols and antigen was retrieved in 0.01 M sodium citrate buffer. Samples were incubated with 3% H_2_O_2_ for 10 min and then blocked in 1% bovine serum albumin in PBS for 1 h. The slides were incubated overnight at 4 °C with Hsp90β, VEGFR1, VEGFR2, CD31 and Ki67 primary antibody. Using the known positive section as the positive criteria, PBS displaces primary antibody as negative criteria. The secondary antibody was followed added with using HRP-Polymer anti-Mouse/Rabbit IHC Kit (Maixin Biotech, China) for 1 h at room temperature. Then, samples were developed with diaminobezidine (Vector Laboratories) reagent and counterstained with hematoxylin and mounted with permount [21].

ImageJ software was used to quantify the MVD based on CD31 staining. The percentage of positively stained area was calculated by using a color deconvolution for separating the staining components (diaminobezidine and hematoxylin) in at least six fields per each sections [21]. The results were presented as percentage of treated group compared to control one. All sections were analyzed and evaluated independently by two pathologists (T.S. and Y.R.L.) at double blinded, and the results were reconfirmed once inconsistent.

### Xenograft tumor model

Approximately 1 × 10^6^ PLC or PLC-Hsp90β cells were injected subcutaneously into the middorsal region of each BALB/c nude mouse. Each group (Control, Hsp90β and NVP-BEP800 group) consisted of eight mice. Tumor-bearing mice began to receive treatment at the time point when the average tumor size reached 100-200 mm^3^. NVP-BEP800 was formulated as a suspension in 0.5% methylcellulose and was given at the dose of 30 mg/kg per day by intragastric administration for 18 days [22]. Solvent buffer at the same volume was used in control and Hsp90β groups. Tumor sizes were measured every 2 days using a caliper and calculated according to a standard formula (length × width^2^ × 0.52) as previously described [23].

### Patient samples

HCC tissue microarrays containing 96 cases were purchased from US Biomax for IHC. Tissue blocks of the tissue array were collected within 5 years. Each single tissue spot on every array slide was individually examined by certified pathologists according to the WHO published standardizations for diagnosis, classification and pathological grade. Both the hospital and the individual consented for each specimen from clinical patients to be included. All tissues were collected under the highest ethical standards; the donor was completely informed and provided consent. All human tissues are collected under HIPPA-approved protocols.

### Statistical analysis

Statistical analyses were performed with GraphPad Prism 6 and SPSS v. 19. Statistically significant differences were calculated by student’s *t*-test, Wilcoxon rank-sum test, Mann–Whitney *U* test, and Pearson’s correlation as appropriate. Values of *p* < 0.05 were considered significant.

## Results

### Hsp90β was associated with HCC angiogenesis

**Fig. 1:**
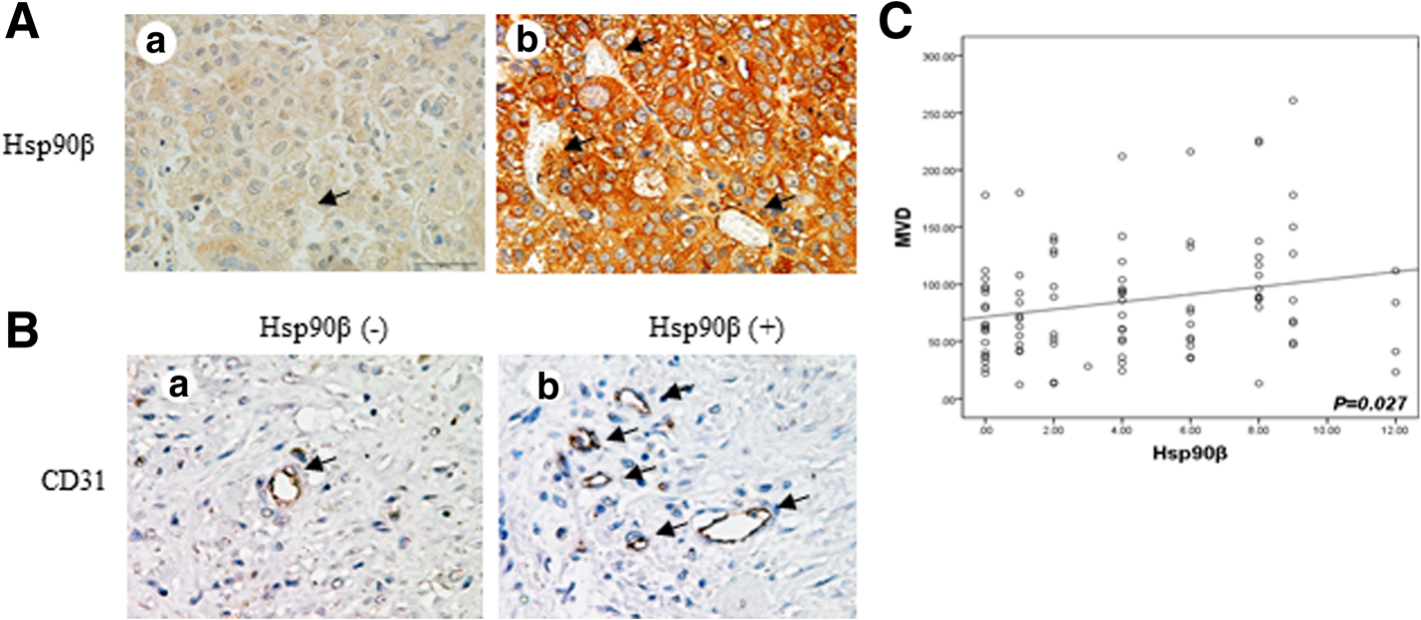
Correlation between Hsp90β and MVD. **a** Representative images of IHC staining for Hsp90β in tissue microarrays of human HCC at different stages (*left*, stage I; *right*, stage IV) showed weak expression in primary cancer tissues (stage I) and strong expression in metastatic tissues (stage IV). *Black arrows* point to microvessels. **b** MVD measured by immunostaining for CD31 in HCC tissue microarrays. MVD was higher in the Hsp90β-positive group than in the Hsp90β-negative group. *Black arrows* point to microvessels. **c** MVD and Hsp90β-positive stainings were quantified and the correlation was analyzed with Spearman correlation method (correlation coefficient: R = 0.226, *P* = 0.027)

Our previous study confirmed that Hsp90β was closely related to the metastasis and recurrence of HCC. Based on the immunohistochemical (IHC) analysis of 96 HCC cases, the expression level of Hsp90β and angiogenesis were higher in HCC with a high degree of malignancy than in HCC with a low degree of malignancy (Fig. 1a). The expression of CD31, a surface marker of neovascular endothelial cells, was detected by immunohistochemical staining of HCC tissues in different stages. Then, microvessel density (MVD) was calculated (Fig. 1b). Pearson correlation and linear regression anaysis showed that the levels of steady state expression of the mRNA of Hsp90β and CD31 was positively correlated (Additional file 1 Fig. S1a). We further analyzed the correlation between Hsp90β expression and MVD. The results showed that Hsp90β was positively correlated with MVD in HCC (*P* = 0.027) (Fig. 1c).

### Hsp90β indicated poor prognosis

**Fig. 2:**
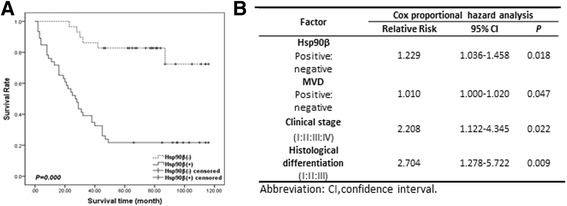
Relationship between Hsp90β and prognosis. **a** Kaplan–Meier curves for the OS of HCC patients with Hsp90β-positive and Hsp90β-negative expression. The Hsp90β-negative group tended to have a longer survival time than the Hsp90β-positive group (*P* = 0.027). **b** Multivariate analyses of factors influencing survival. Cox proportional hazard model analysis was performed with clinicopathological factors (clinical stage, *P* = 0.022; histological differentiation, *P* = 0.009), Hsp90β expression (positive/negative, *P* = 0.018), and MVD (positive/negative, *P* = 0.047)

Kaplan–Meier survival analysis revealed that patients with high levels of Hsp90β expression had significantly lower overall survival (OS) compared with those with low Hsp90β expression (*P* = 0.000) (Fig. 2a). Cox proportional hazards model analysis was performed on the basis of clinicopathological factors, Hsp90β expression (high/low), and MVD presence (positive/negative). Multivariate analysis showed that high Hsp90β expression, MVD, advanced clinical stage, and histological differentiation were indicators of poor prognosis (*P* = 0.018, 0.047, 0.022, and 0.009, respectively) (Fig. 2b).

### Correlation between Hsp90β and EGFRs

**Fig. 3:**
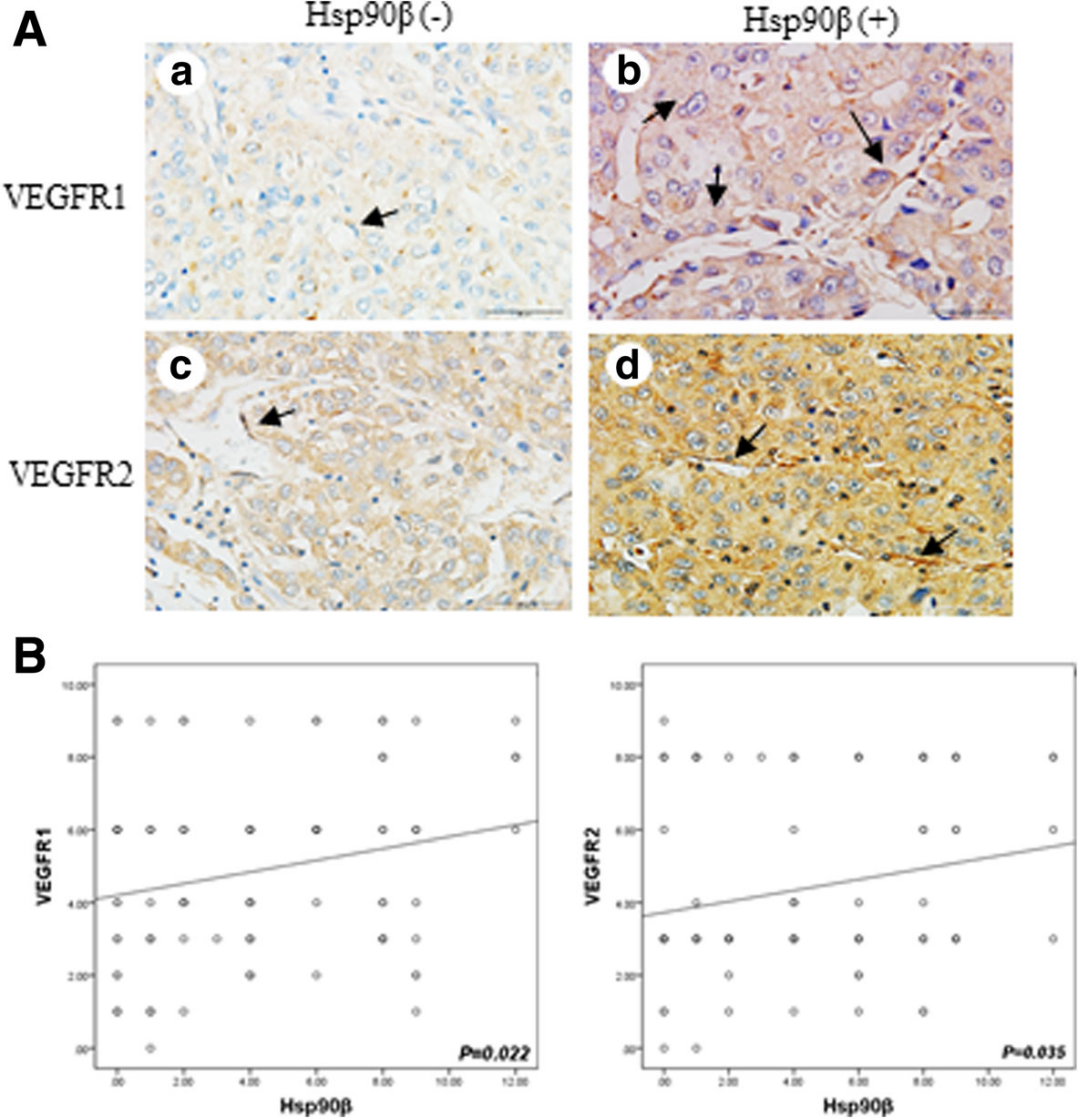
VEGFR1 and VEGFR2 expression in Hsp90β-negative (Hsp90β-) and Hsp90β-positive (Hsp90β+) HCC tissues. **a** *a* and *c*, VEGFR1 and VEGFR2 expression in Hsp90β- HHC group; *b* and *d*, VEGFR1 and VEGFR2 expression in Hsp90β + HCC group (original magnification × 400). *Black arrows* point to microvessels. **b** Correlation analysis between Hsp90β and VEGFR1 (*P* = 0.022) and VEGFR2 (*P* = 0.035)

We analyzed 96 liver cancer cases and found that the expression levels of Hsp90β, VEGFR1, and VEGFR2 in liver tumor tissues were correlated with the pathological grade and clinical stage of the tumor (Table 1). Immunohistochemical staining showed that HCC patients with high Hsp90β expression levels also highly expressed VEGFR1 and VEGFR2. Additionally, VEGFR1 and VEGFR2 expression levels were higher in the endothelial cells surrounding vessels (Fig. 3a). Correlation analysis showed that Hsp90β was associated with VEGFR1 and VEGFR2 expression (*P* = 0.022 and *P* = 0.035, respectively) (Fig. 3b). The correlation studies of Hsp90β and VEGFR1, VEGFR2 mRNA levels were consistent with those of IHC evaluation (Additional file 1 Fig. S1b, c).

**Table 1:** Correlation between Hsp90β, VEGFR1, VEGFR2, and primary tumor characteristics

Variant	Hsp90β		X^2^	*P*	VEGFR1		X^2^	*P*	VEGFR2		X^2^	*P*
	Low	High			Low	High			Low	High		
Age (year)												
≤60	204	53	0.001	0.973	67	12	0.701	0.403	60	19	0.918	0.338
>60	39	10			13	4			11	6		
Sex												
M	202	58	3.128	0.077	74	13	1.986	0.159	64	23	0.075	0.784
F	41	5			6	3			7	2		
Histological differentiation												
≤II	180	38	4.621	0.032^*^	62	16	4.431	0.035^*^	61	17	3.895	0.048^*^
>II	63	25			18	0			10	8		
Stage												
≤II	148	47	4.061	0.044^*^	46	14	5.120	0.024^*^	48	12	3.032	0.082
>II	95	16			34	2			23	13		

*Significantly different, *P* <0.05

### Hsp90β stimulated endotheliocytes to proliferate and accelerate neovascularization depending on VEGFR expression

**Fig. 4:**
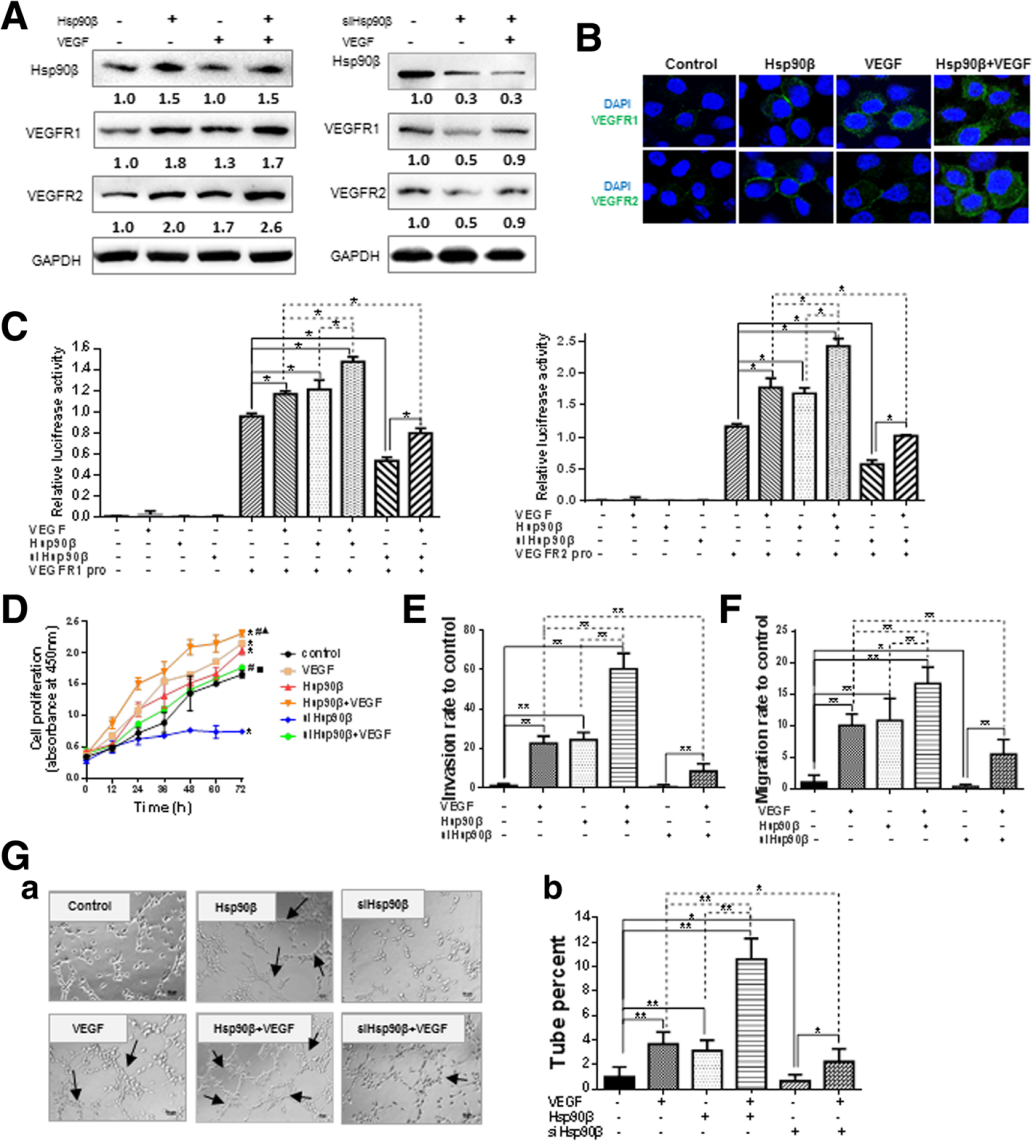
Hsp90β promotes endotheliocyte proliferation and accelerates neovascularization. **a** Western blot analysis showed VEGFR1 and VEGFR2 expression levels in HUVEC cells overexpressed or knocked down Hsp90β and/or under VEGF treatment. **b** Immunofluorescence of VEGFR1 and VEGFR2 expression in HUVEC cells overexpressed Hsp90β alone, VEGF treatment alone or overexpressed Hsp90β under VEGF treatment. **c** HUVEC cells were transiently transfected with VEGFR1- or VEGFR2- dependent reporter gene plasmids. Luciferase activity was measured when cells overexpressed or knocked down Hsp90β and/or under VEGF treatment. **d** Growth curve of HUVEC cells overexpressed or knocked down Hsp90β and/or under VEGF treatment, measured by SRB assay. (*, vs control group, *P* < 0.05; #, vs VEGF group, *P* < 0.05; ▲, vs Hsp90β group, *P* < 0.05; ■, vs siHsp90β group, *P* < 0.05) **e** Transwell assays indicated invasion ability when HUVEC cells overexpressed or knocked down Hsp90β and/or under VEGF treatment. **f** Migration ability increased when HUVEC cells overexpressed or knocked down Hsp90β and/or under VEGF treatment. **g** In vitro assay for vascular mimicry of HUVEC cells in three-dimensional culture at 12 h. *a*, Representative graphs; black arrows point to tubes. *b*, Tube formation quantification was analyzed after HUVEC cells overexpressed or knocked down Hsp90β and/or under VEGF treatment. *P < 0.05, **P < 0.01

VEGFRs are major receptors on endothelial cells that are involved in multiple signaling pathways, including the induction of mitogenesis, migration, invasion, and differentiation in neoangiogenesis [24]. Sanderson et al. [18] reported that VEGFR2 is a client protein of Hsp90 and they can form a complex in HUVECs to promote endothelial cell migration; they also reported that VEGFR2 deletion mutants are unable to associate with Hsp90 [19, 25]. To elucidate the underlying mechanism of Hsp90β in inducing angiogenesis, we evaluated the effects of Hsp90β on VEGFRs expression. Our results showed that as with VEGF treated HUVEC cells, VEGFR1 and VEGFR2 expression levels also increased after Hsp90β overexpression (Fig. 4a). This result was validated by immunofluorescence staining (Fig. 4b). Whereas, VEGFR1 and VEGFR2 expression levels decreased after Hsp90β knocked down. When the HUVEC cells simultaneously treated with siHsp90β and VEGF, VEGFR1 and VEGFR2 expression levels increased compared with alone Hsp90β knocked down group (Fig. 4a). Furthermore, we used a dual-luciferase report system to determine the effect of Hsp90β on the promoter activities of VEGFR1 and VEGFR2. The results showed that Hsp90β and/or VEGF increased VEGFR1 and VEGFR2 promoter activities, Hsp90β knockdown decreased VEGFR1 and VEGFR2 promoter activities and VEGF released the inhibition effect of Hsp90β knocked down on VEGFRs promoter activities (Fig. 4c). In addition, Hsp90β promoted HUVEC proliferation and Hsp90β knockdown inhibited HUVEC proliferation. VEGF treatment counteracted the inhibitory effect of knocked down Hsp90β (Fig. 4d). Then, we performed Transwell chamber (with or without Matrigel on the filters) invasion and migration assays to examine the effects of Hsp90β on HUVEC invasion and migration. The number of HUVECs that migrated through the filters showed that cell invasion and migration significantly increased after Hsp90β overexpression and/or VEGF treatment. The cell invasion and migration decreased obviously when Hsp90β knocked down and VEGF released the inhibition effect of knocked down Hsp90β (Fig. 4e and f).

In vitro, endothelial cells can form a three-dimensional tube in Matrigel culture. To examine the effects of Hsp90β on HUVEC tube formation, we seeded HUVECs that were transfected with Hsp90β, Hsp90β siRNA, and/or treated with VEGF for 48 h on Matrigel. The results showed that Hsp90β significantly enhanced HUVEC tube formation, whereas Hsp90β silencing inhibited HUVEC tube formation. VEGF increased the effect of Hsp90β on angiogenesis and knockdown of Hsp90β obviously decreased VEGF-induced tube formation (Fig. 4g). Our results showed that Hsp90β affected in vitro VEGFRs promoter activities, VEGFRs expression, and HUVEC proliferation, migration, invasion, tube formation, and angiogenesis.

### Hsp90 inhibitor blocked the proliferation, migration, invasion, and tube formation of endothelial cells

**Fig. 5:**
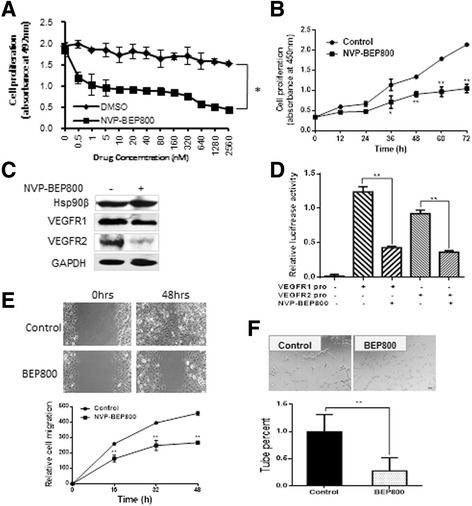
NVP-BEP800 inhibited endothelial cell proliferation, migration, invasion, and tube formation. **a** Incubation with different concentrations of NVP-BEP800 for 48 h inhibited HUVEC cell proliferation. **b** Growth curve of HUVEC cells incubated with 2 μM NVP-BEP800, as measured by SRB assay. **c** Western blot analysis showed that treatment with 2 μM NVP-BEP800 down-regulated VEGFR1 and VEGFR2 expression in HUVEC cells. **d** HUVEC cells were transiently transfected with VEGFR1- or VEGFR2- dependent reporter gene plasmids and treated with 2 μM NVP-BEP800. After 24 h, the cells were lysed and luciferase activity was measured. **e** The wound healing ability of HUVEC cells was assessed after 48 h of NVP-BEP800 treatment. DMSO treatment was used as the standardized control for quantification. **f** In vitro assay for vascular mimicry of HUVEC cells treated with NVP-BEP800 for 48 h in three-dimensional culture. NVP-BEP800 inhibited cord formation of HUVEC cells. *P < 0.05, **P < 0.01

The ability of Hsp90 inhibitors, such as geldanamycin and its derivatives, to suppress endothelial cell proliferation has been previously described [26]. However, the effect of NVP-BEP800, a new Hsp90 inhibitor, on endothelial cells has not been reported. To determine the direct cytotoxic effects of NVP-BEP800 on endothelial cells, we conducted an in vitro cytotoxicity experiment. The different concentrations of NVP-BEP800 were cytotoxic to HUVECs when given as a treatment for 48 h (Fig. 5a). In addition, the toxicity of NVP-BEP800 in normal human liver cells (HHL-5) and various HCC cell lines (SMMC-7721, PLC-PRF-5, BEL-7402) were also measured. The results showed that NVP-BEP800 has no significant toxicity in HHL-5, and inhibited the proliferation of ﻿HCCs (Additional file 2 Fig. S2). HUVECs in log-phase growth were measured over 72 h and showed that NVP-BEP800 (2 μM) inhibited the proliferation of human endothelial cells (Fig. 5b). There was no obvious effect on death and apoptosis of HUVECs under 2 μM NVP-BEP800 treatment for 48 h (Additional file 3 Fig. S3). Western blot analysis showed that VEGFR1 and VEGFR2 expressions decreased under 48 h of 2 μM NVP-BEP800 treatment (Fig. 5c). Furthermore, we analyzed the effect of NVP-BEP800 on VEGFRs promoter activities. The results of the dual-luciferase reporter assay showed that NVP-BEP800 significantly decreased VEGFR1 and VEGFR2 promoter activities (Fig. 5d). In addition, a scratch wound assay showed that Hsp90 inhibitors prevented HUVEC haptotaxis. Control cells completed wound closure after 48 h. By contrast, cells treated with 2 μM NVP-BEP800 failed to complete wound closure within 48 h (Fig. 5e). To test the effect of NVP-BEP800 inhibition on HUVEC tube formation, HUVECs were treated with 2 μM NVP-BEP800 for 24 h and then seeded on Matrigel for 12 h. The results showed that NVP-BEP800 significantly decreased tube formation compared with control cells (Fig. 5f). We evaluated the tube formation in HUVECs that knocked down Hsp90α or Hsp90β under NVP-BEP800 treatment. The results showed that compared to Hsp90α knocked down﻿ group, the tube formation in siHsp90α+NVP-BEP800 group were significantly reduced. While there was no statistically significant difference in Hsp90β knocked down group and siHsp90β+NVP-BEP800 group (Additonal file 4 Fig. S4). It is indicated that the inhibition of endothelial cell-dependent tumor angiogenesis of NVP-BEP800 was primarily by inhibiting the activity of Hsp90β. ﻿ In addition, 17-Allylamino-17-demethoxygeldanamycin (17-AAG) were compared with NVP BEP800 on the proliferation and tube formation of HUVECs (Additonal file 5 Fig. S5). The results showed that 17-AAG inhibited cell proliferation, while the inhibition effect of NVP-BEP800 was stronger than 17-AAG on tube formation.

### Hsp90β promoted angiogenesis and Hsp90 inhibitor suppressed angiogenesis in vivo

**Fig. 6:**
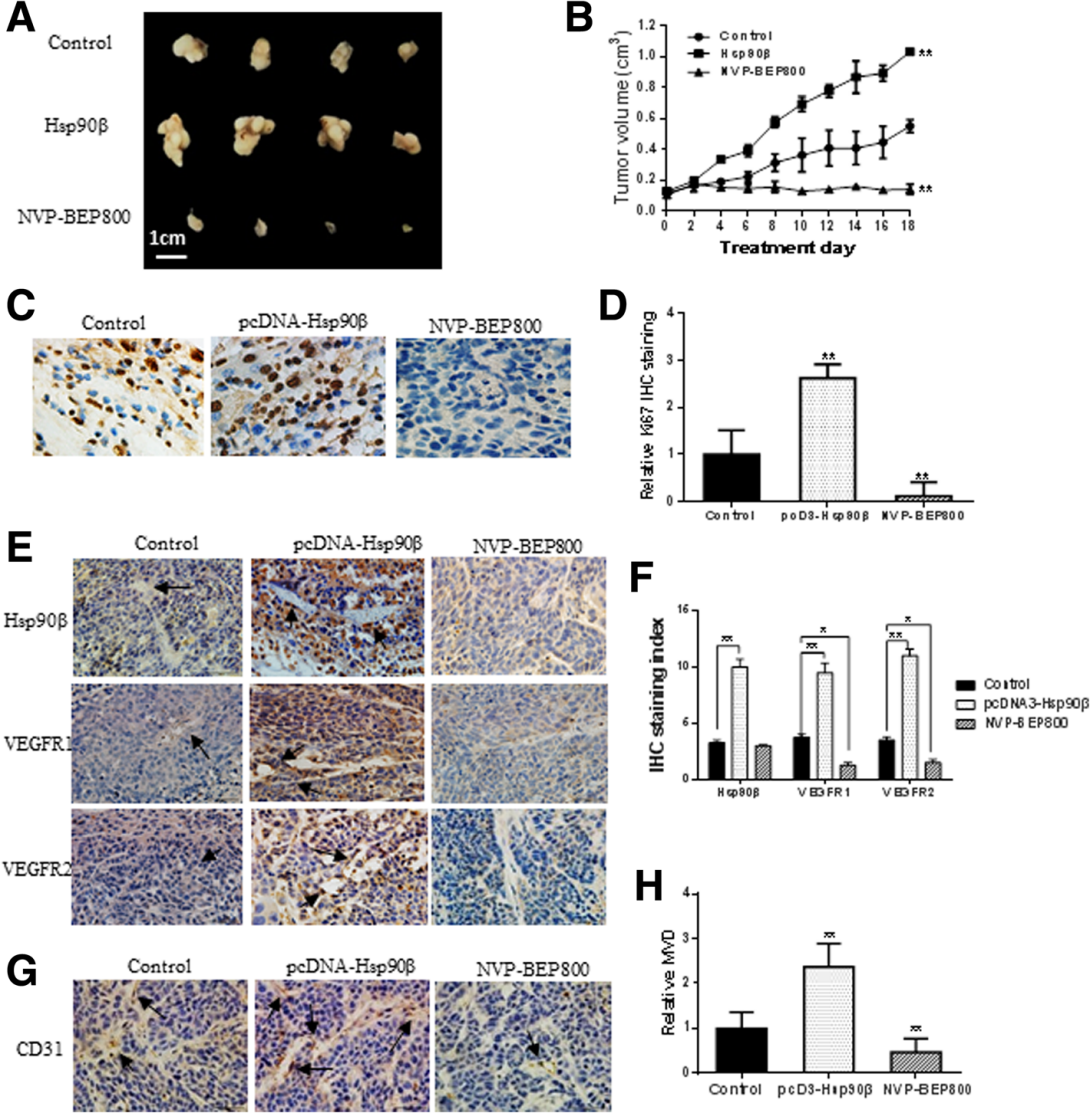
Immunohistochemical analysis of VEGFRs expression and tumor vascularization in a HCC xenograft model. **a** Control group mice subcutaneously received PLC cell suspensions. Hsp90β group mice subcutaneously received a suspension of PLC cells transfected with pcDNA-Hsp90β plasmids. NVP-BEP800 group mice subcutaneously received a suspension of PLC cells and were treated by intragastric administration with NVP-BEP800 (30 mg/kg/day). **b** Tumor sizes were measured every 2 days and tumor volumes were calculated. **c**, **d** To check the effect of Hsp90β and NVP-BEP800 on cell proliferation, immunohistochemical analysis of Ki67 was performed with the tumor issues. The intensity of Ki67-positive cell was calculated with Image J software. **e**, **f** VEGFR1 and VEGFR2 expression in tumor tissue of the control group, Hsp90β group, and NVP-BEP800 group mice. **g**, **h** Analysis of MVD based on CD31 staining of tumor tissue by Image J software showed that vessel number significantly increased in the Hsp90β group and that vessel number decreased in the NVP-BEP800 group. *P < 0.05, **P < 0.01

We demonstrated that Hsp90β promoted endothelial cell proliferation, migration, and tube formation and that the Hsp90 inhibitor attenuated VEGFR levels and inhibited tube formation in vitro. Then, we constructed a nude mice subskin model that was transplanted with human liver cancer. Control group mice subcutaneously received PLC cell suspensions. Hsp90β group mice subcutaneously received a suspension of PLC cells transfected with pcDNA-Hsp90β plasmids. NVP-BEP800 group mice subcutaneously received a suspension of PLC cells. When the tumors volume reached 100-200 mm^3^, NVP-BEP800 group mice were treated with NVP-BEP800 (30 mg/kg/daily) by intragastric administration for 18 days and solvent buffer at the same volume was used in control and Hsp90β groups. The results showed that compared with the control group, Hsp90β promoted the liver tumor growth, and NVP-BEP800 inhibited tumor growth in vivo (Fig. 6a, b). To further check the effect of Hsp90β and NVP-BEP800 on cell proliferation, immunohistochemical analysis of Ki67 was performed with the tumor issues (Fig. c, d). Also, we demonstrated that VGFR1 and VEGFR2 expression levels increased in cells overexpressing Hsp90β, but decreased after NVP-BEP800 treatment (Fig. 6e, f). Immunohistochemical detection revealed that CD31 expression and MVD in the Hsp90β-overexpressing group were significantly higher than those in the control group, but decreased in the presence of NVP-BEP800 (Fig. 6g, h). There was no significant difference about blood vessel tissue between control and NVP-BEP800 group by analysis mice liver and lung pathological section using hematoxylin and eosin staining (Additonal file 6 Fig. S6). Our data suggested that Hsp90β contributed to endothelial cell-dependent angiogenesis.

## Discussion

Angiogenesis is critical to tumor growth, invasion, and metastasis, especially during the early stages of tumor development. Our previous work demonstrated that Hsp90β is a marker protein for detecting liver cancer malignancy. Based on the analysis of 96 liver cancer cases, we demonstrated that Hsp90β was up-regulated in HCCs with a high degree of malignancy. Especially, in HCC tissues, Hsp90β expression in vascular endothelial cells also significantly increased. We examined CD31-positive endothelial cell-dependent vascular density and found that Hsp90β expression was positively correlated with MVD. The survival time of Hsp90β-negative HCC patients was longer than that of Hsp90β-positive HCC patients. Cox regression analysis showed that Hsp90β expression and MVD, as well as the clinical stage and pathological grade of the tumor, are risk factors that determine the survival of HCC patients. Furthermore, we found that VEGFRs were highly expressed in HCC tissues with high Hsp90β expression in endothelial cells. Statistical analysis showed that Hsp90β expression was correlated with VEGFRs.

In vitro, Hsp90β promoted VEGFR1 and VEGFR2 promoter activities. Furthermore, Hsp90β promoted VEGFR expression in endothelial cells. VEGFRs are crucial to tumor angiogenesis, promote the proliferation of endothelial cells, and increase vascular permeability [27]. The stimulating effect of Hsp90β was more obvious on VEGFR2 promoter activity than on VEGFR1 promoter activity, which indicated that Hsp90β mainly acts on VEGFR2. After Hsp90β overexpression, endothelial cell proliferation, invasion, migration, and tubular structure formation increased. The promoting effect was more apparent when VEGF treatment was simultaneously performed. This may be due to Hsp90β promoting VEGFRs expression by stimulating VEGFRs promoter activities, and after VEGF treatment, more receptors were induced to combine with VEGF and lead to active a series of downstream pathways. Hsp90β knockdown decreased the ability of endothelial cells to form tube structures. Tube formation of simultaneous Hsp90β knockdown and VEGF treatment group was decreased obviously compared with the VEGF-only treated group. This finding indicated that knocked-down Hsp90β counteracted the tube formation induced by VEGF. Compared with Hsp90β knockdown group, the simultaneous Hsp90β knockdown and VEGF treatment group had increased tube formation. This finding indicated that VEGF can release the inhibition caused by Hsp90β knockdown in HUVEC cells. Also, it means that VEGF can release the inhibition on angiogenesis caused by Hsp90β knockdown. Intratumoral MVD and VEGFRs expression increased in the nude mice that were subcutaneously tumor burdened with Hsp90β-overexpressing HCC cells.

Based on the role of Hsp90β in angiogenesis, we sought to identify Hsp90β inhibitors as antitumor agents by anti-angiogenesis. Hsp90 inhibitors, such as NVP-AUY922 [28], VER49009/VER50589 [29], and geldanamycin derivatives, including 17-AAG and 17-DMAG [26, 30–32], are used as antitumor and antiangiogenesis drugs. The direct effect of NVP-BEP800, a new Hsp90β inhibitor, on anti endothelial cell-dependent angiogenesis has never been reported. The antiangiogenic activities of Hsp90β inhibitors directly affected endothelial cells. NVP-BEP800 inhibited vascular endothelial cell proliferation. This inhibitory effect was dose-dependent. Endothelial cell migration, invasion, and tube formation directly affected the angiogenic process. There was reported that Hsp90 forms a complex with VEGFR2, but VEGFR2 deletion mutants cannot associate with Hsp90 [19, 25]. Interestingly, our findings showed that NVP-BEP800 decreased VEGFR1 and VEGFR2 expression levels by inhibiting VEGFR1 and VEGFR2 promoter activities. Our in vitro studies indicated that tumors in mice treated with NVP-BEP800 showed decreased VEGFRs expression and reduced microvascular density. In addition, no significant difference in the blood vessel tissues of liver and lung was observed between the normal and NVP-BEP800 treated mice. So we hold that the NVP-BEP800 may only affect the tumor neovascularization process and may have no significant effect on the blood vessels of the normal tissues.

In conclusion, our data illustrated that Hsp90β promoted endothelial cell-dependent tumor angiogenesis by increasing VEGFRs expression. Moreover, NVP-BEP800, a Hsp90 inhibitor, exerted antiangiogenic activity when administered orally. Moreover, it is reported that NVP-BEP800 used as an antineoplastic agent, via decreasing Hsp90 client proteins of downstream [22]. Therefore, Hsp90 inhibitors may target multiple pivotal points in both tumor and endothelial cell survival, proliferation, and invasion to provide a combinatorial approach to attack multiple features of malignant phenotypes [18]. Hsp90 inhibitors may be used as a therapeutic strategy to inhibit tumor angiogenesis by decreasing endothelial cell progression and metastasis.

## Conclusions

Hsp90β induced endothelial cell-dependent tumor angiogenesis by activating VEGFRs transcription. We provided new insight into the regulatory mechanism of tumor angiogenesis by exploring the role of Hsp90β, which may also help develop a therapeutic strategy for tumor treatment through the inhibition of endothelial cell progression and metastasis.

## Additional files

Additional file 1: Figure S1. Comparative distribution of the Hsp90β and CD31 (a), VEGFR1 (b), VEGFR2 (c) expression levels. (TIF 438 kb)

Additional file 2: Figure S2. Inhibition of HHL-5 (a), SMMC-7721 (b), PLC-PRF-5 (c), and BEL-7402 (d) cells via NVP-BEP800 treatment. (TIF 647 kb)

Additional file 3: Figure S3. HUVEC cell death and apoptosis results under NVP-BEP800 treatment. (TIF 1300 kb)

Additional file 4: Figure S4. (a) Western blot analysis of Hsp90α and Hsp90β expression in HUVEC cells with knocked down Hsp90α or Hsp90β under NVP-BEP800 treatment. (b) Tube. (TIF 1105 kb)

Additional file 5: Figure S5. Inhibition of HUVEC cell proliferation (a) and tube formation (b) via 17-AAG and NVP-BEP800 treatments. (TIF 824 kb)

Additional file 6: Figure S6. HE staining of mice liver and lung pathological sections in the control and NVP-BEP800 groups. (TIF 4229 kb)

## Abbreviations

HCC: Hepatocellular carcinoma
Hsp90β: Heat shock protein 90beta
MVD: Microvascular density
SEAP: Secreted alkaline phosphatase
VEGFR: Vascular endothelial growth factor receptor
