# Supplementary figures and images for: Hsp90β promoted endothelial cell-dependent tumor angiogenesis in hepatocellular carcinoma
Source: Mol Cancer. 2017 Mar 31;16:72. doi: 10.1186/s12943-017-0640-9 (PMC5374580; doi:10.1186/s12943-017-0640-9)

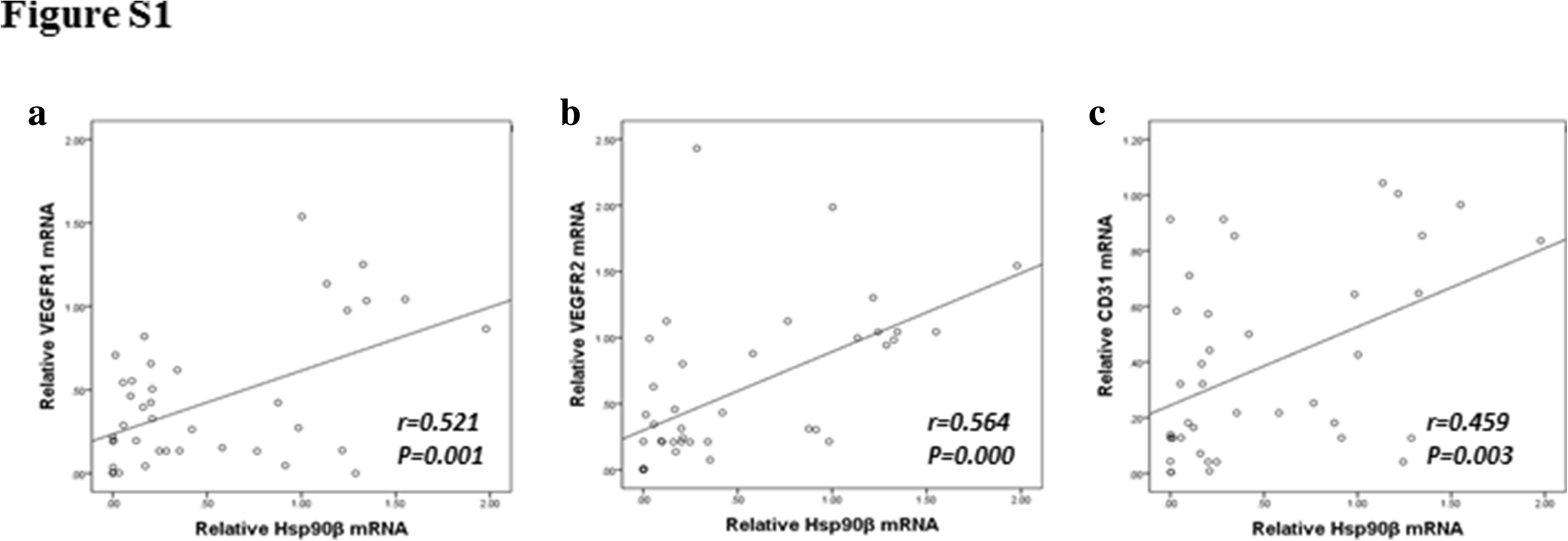

Supplement: Supplementary file 1 — Comparative distribution of the Hsp90β and CD31 (a), VEGFR1 (b), VEGFR2 (c) expression levels. (TIF 438 kb) [file 12943_2017_640_MOESM1_ESM.tif]

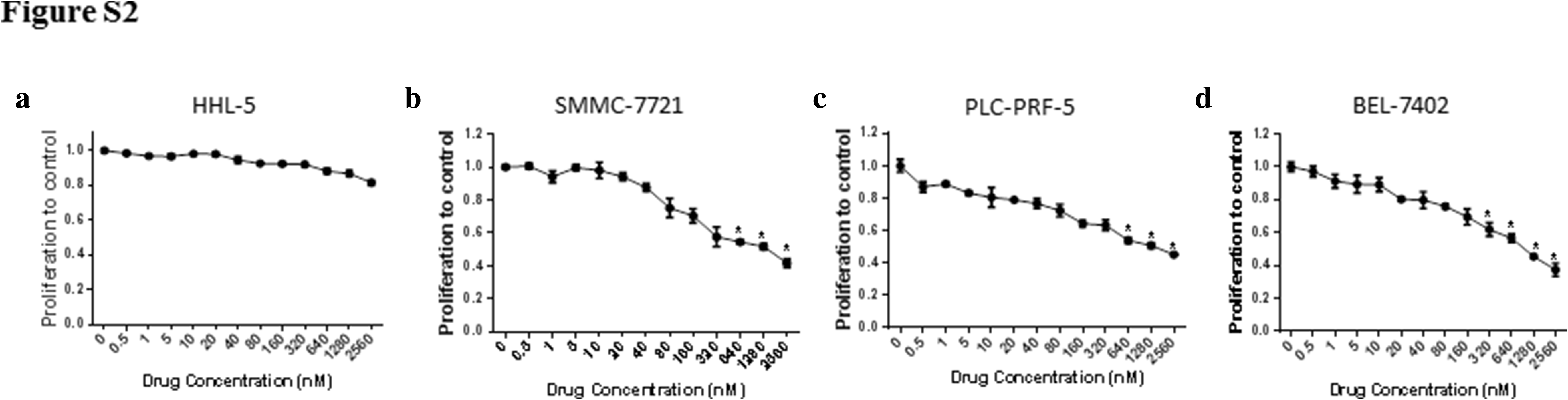

Supplement: Supplementary file 2 — Inhibition of HHL-5 (a), SMMC-7721 (b), PLC-PRF-5 (c), and BEL-7402 (d) cells via NVP-BEP800 treatment. (TIF 647 kb) [file 12943_2017_640_MOESM2_ESM.tif]

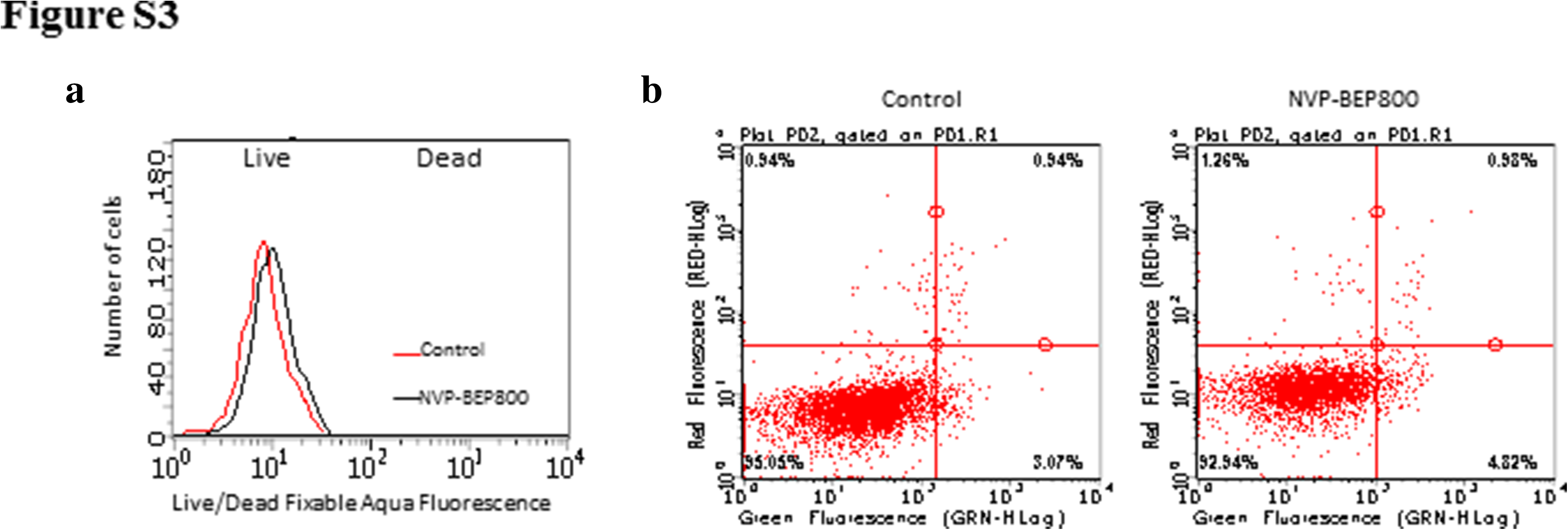

Supplement: Supplementary file 3 — HUVEC cell death and apoptosis results under NVP-BEP800 treatment. (TIF 1300 kb) [file 12943_2017_640_MOESM3_ESM.tif]

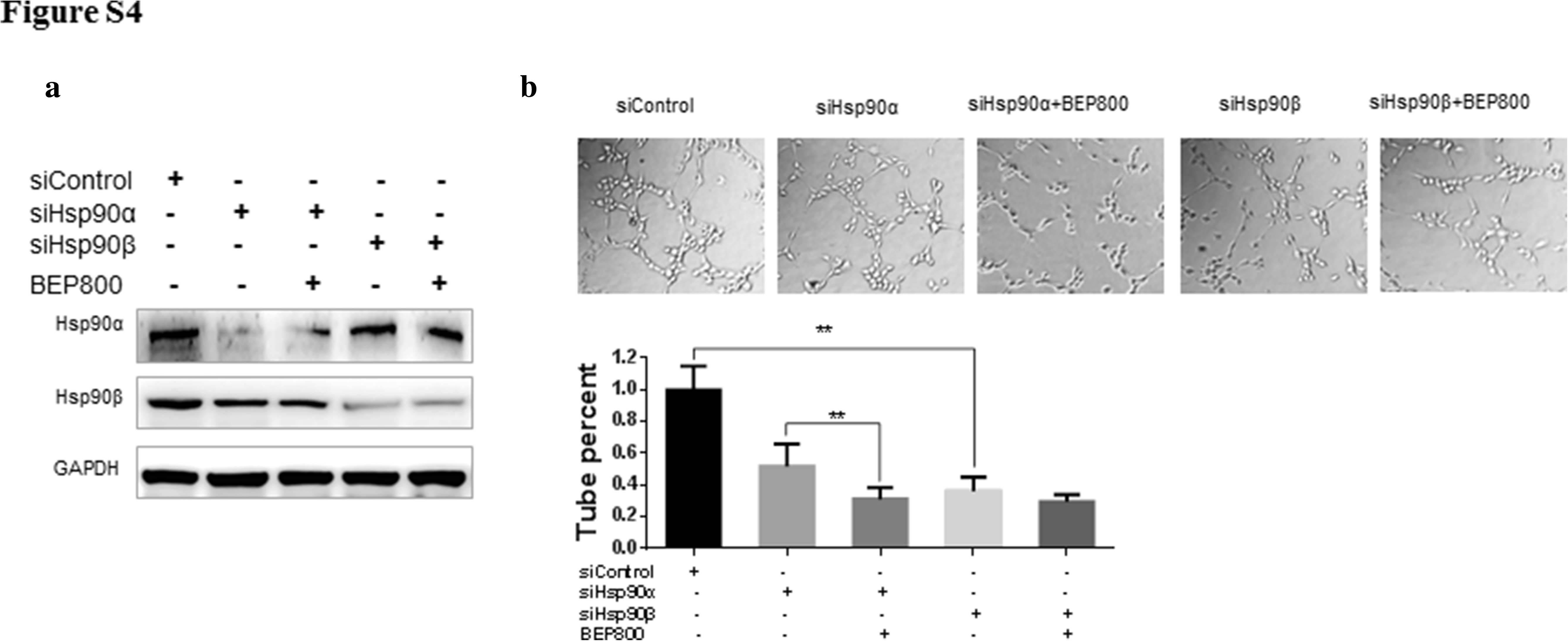

Supplement: Supplementary file 4 — (a) Western blot analysis of Hsp90α and Hsp90β expression in HUVEC cells with knocked down Hsp90α or Hsp90β under NVP-BEP800 treatment. (b) Tube. (TIF 1105 kb) [file 12943_2017_640_MOESM4_ESM.tif]

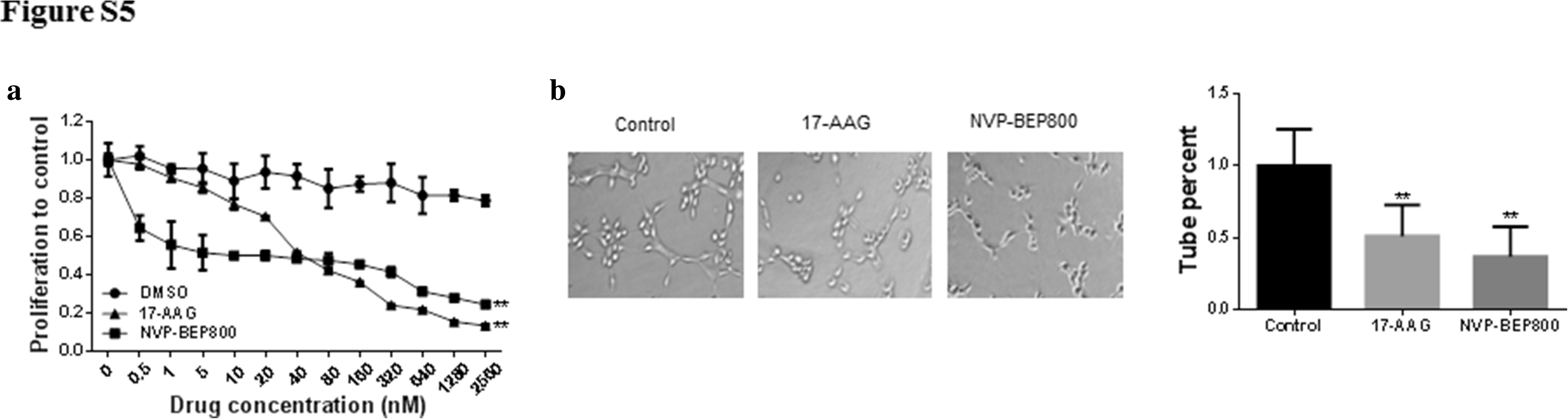

Supplement: Supplementary file 5 — Inhibition of HUVEC cell proliferation (a) and tube formation (b) via 17-AAG and NVP-BEP800 treatments. (TIF 824 kb) [file 12943_2017_640_MOESM5_ESM.tif]

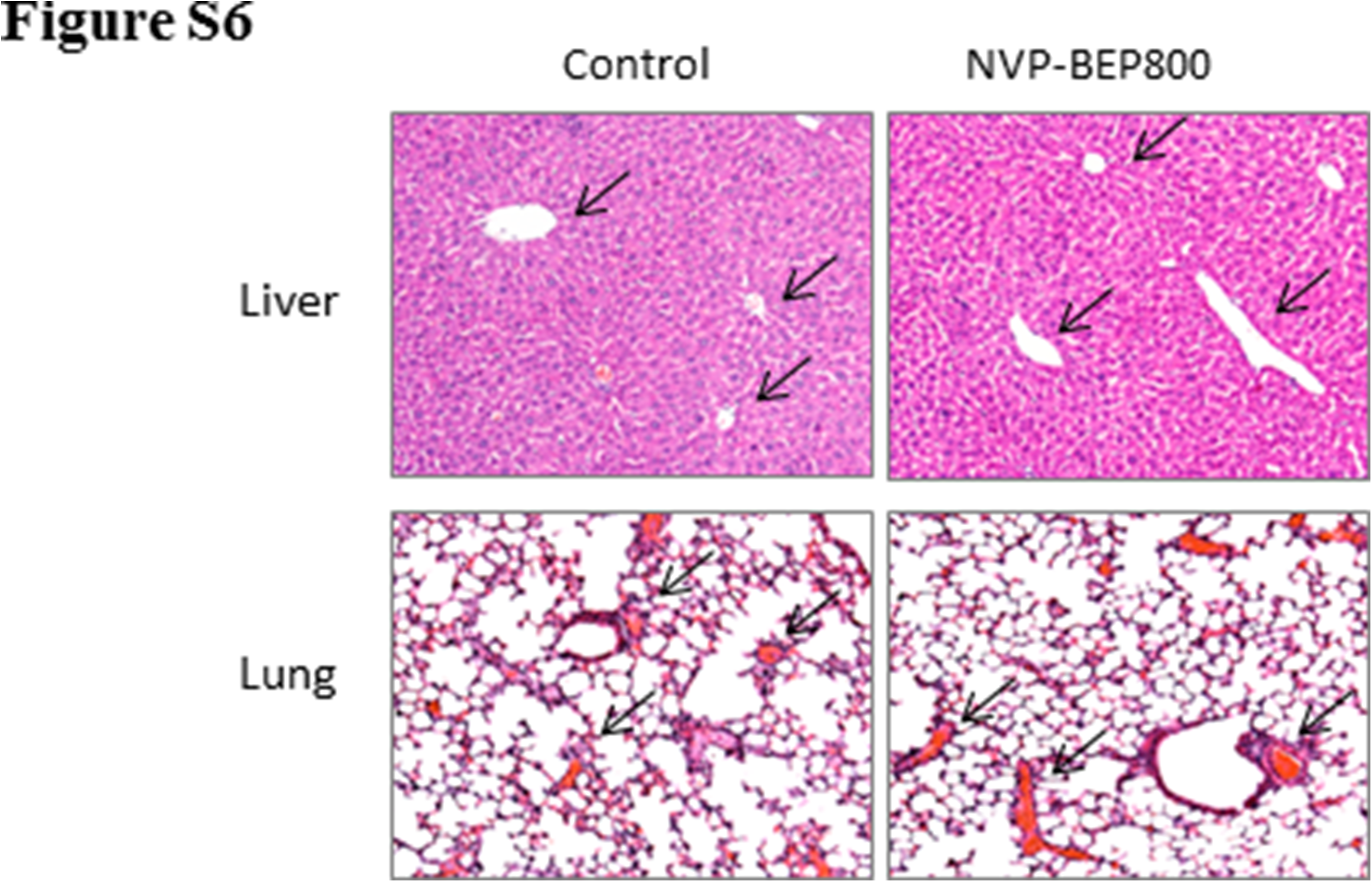

Supplement: Supplementary file 6 — HE staining of mice liver and lung pathological sections in the control and NVP-BEP800 groups. (TIF 4229 kb) [file 12943_2017_640_MOESM6_ESM.tif]
